# Understanding avoidance of medical surveillance for hand-arm vibrations in Sweden: a mixed-methods approach

**DOI:** 10.3389/fpubh.2026.1789291

**Published:** 2026-03-31

**Authors:** Carl Antonson, Carita Håkansson, Frida Thorsén

**Affiliations:** 1 Division of Occupational and Environmental Medicine, Department of Laboratory Medicine, Lund University, Lund, Sweden; 2 Center for Primary Health Care Research, Department of Clinical Sciences Malmö, Lund University, Malmö, Sweden

**Keywords:** compliance, exposure, hand-arm vibration syndrome, mixed methods, participation rate, qualitative research, surveillance system

## Abstract

**Introduction:**

Hand-arm vibration syndrome (HAVS) is a chronic disease to vessels and nerves caused by vibrations. In Sweden, the medical check-up for vibrations (MCV) implements the EU surveillance system to detect early cases. No earlier research has shown participation in MCVs, nor why MCVs is avoided. We aimed to improve the understanding on avoidance of MCVs among vibration-exposed workers.

**Methods:**

We used a mixed method. Participation was estimated from sales data registries from the three largest occupational health services (OHS) in Sweden, comprising 50% of the market share. Exposure figures for the quantitative estimates came from both public data and exposure-data from a meta-analysis. The qualitative aim was explored through strategic telephone interviews with 26 workers with known symptoms of HAVS using hermeneutical methods.

**Results:**

MCV-participation has increased five-fold from 2016–2022. In 2020–2022, 11,643 MCVs were performed in the three OHS, giving a national estimate of 22,000 MCVs. We estimated the number of exposed workers to be 218,000 by exposure, and 400,000 by statistical sources only. MCVs were mainly avoided because they are not offered by employers despite being mandatory, and due to fear of the socio-economic consequences of a HAVS diagnosis. Other reasons included bullying, inconvenience, perceived lack of necessity, and limited awareness.

**Discussion:**

Only 5%−10% of exposed Swedish workers attend an MCV despite its importance. A cohesive and transparent process is proposed to increase the low participation rate to avoid the common, chronic, and debilitating injuries from HAVS.

## Introduction

Hand-arm vibration syndrome (HAVS) is the most commonly reimbursed occupational disorder in Swedish men, representing more than 1/3 of the cases. In women, it accounts for 6% of reported occupational disorders. The number of reported cases has increased over time (1–3). HAVS is characterized by injuries of nerves and vessels in the hands and arms. It is caused by exposure to vibrations and worsens with increased exposure time and magnitude of vibration (4). As HAVS in the more advanced stages is considered a chronic condition, finding workers with incipient HAVS is crucial, and prevention is mandated (5–8).

In Sweden, the employer bears the primary responsibility for the work environment, as well as for workplace rehabilitation and accommodation. Under the Work Environment Act [(45), p. 1160, Ch. 3] employers are required to take all necessary measures to prevent employees from being exposed to ill health or accidents. Employers must also ensure that workplace adaptation and rehabilitation activities are appropriately organized in accordance with their obligations under both the Work Environment Act and the Swedish Social Insurance Code (Socialförsäkringsbalken, Ch. 30). Furthermore, employers are responsible for ensuring access to occupational health services (OHS) when required by working conditions. Such services are defined as an independent expert resource in the fields of occupational health and rehabilitation. Where sufficient internal competence is lacking, the employer must engage external OHS.

According to the Swedish provision AFS 2023:15, all employers are required to provide a Medical Check-Up in Worklife for Vibrations (MCV) to each worker exposed to hand–arm vibration when specific criteria are met. This obligation applies when the daily action value of 2.5 m/s^2^ A(8) is exceeded, when the nature of the exposure reasonably suggests a risk of ill health, disease, or injury—even if the action value is not surpassed—such as in dentistry (9) or work involving percussive impact tools like riveting hammers (10), or when vibration injuries, or suspected vibration injuries, have occurred in another worker with comparable exposure.

The MCV must be conducted by a physician and offered prior to the start of vibration-exposed work, with follow-up examinations every 3 years. If early signs of HAVS are detected, more frequent assessments may be recommended. National guidelines have been issued (11, 12) recommending what procedures should be included to fulfill the requirements of the examination (13).

According to Nilsson et al. (7) approximately 400,000 Swedish workers, the majority male, are exposed to vibrations for at least 25% of their work day and would thus likely qualify for the inclusion criteria for participation in an MCV according to a report from EU-OSHA (14). It is not known how many employers arrange such examinations, nor how many workers participate in them. Clinically, there is a suspicion that the participation is poor (15), but to the best of our knowledge, there are still no data on participation in MCVs published.

Moreover, we do not know why vibration-exposed individuals chose to attend, or to abstain from, the MCVs. It has been reported that machismo can lead to a reluctance in reporting HAVS symptoms to health professionals, leading to more advanced HAVS symptoms (16). Delayed access to health care for HAVS also appears to be due to workers minimizing symptoms and their ignorance of the condition, as well as primary health-care providers' failure to diagnose and connect symptoms to work (17). However, the topic remains largely unexplored.

This study provides novel empirical evidence on the real-world implementation of an occupational disease surveillance programme for HAVS. As to date there is little information of utilization and effectiveness of such programmes and this study addresses, and starts filling, this significant gap in knowledge.

### Aims

The overall aim of the study was to improve the understanding of avoidance of medical check-ups (MCVs) among vibration-exposed workers.

The **quantitative aim** was to assess the extent of the avoidance problem by estimating the proportion of vibration-exposed workers at risk of developing HAVS participating in the Swedish medical surveillance system.

The **qualitative aim** was to explore why workers with symptoms or signs of HAVS avoid MCVs and to identify factors that could enhance participation.

## Materials and methods

This study is a mixed method study with a quantitative approach for the participation rate and a qualitative, hermeneutical approach for the reasons why participation was avoided and what factors could mitigate this.

### Quantitative data collection

#### Number of performed MCVs

To estimate the number of MCVs performed, data on all sold MCVs were collected from Sweden's three largest OHS providers, Falck (previously Previa, rebranded in 2022), Avonova, and Feelgood, which together represent half of the national OHS market (personal communication, Peter Munck af Rosenschöld, CEO of the Swedish OHS branch organization). These providers primarily serve large and medium-sized companies in the construction and manufacturing sectors, which have the highest risk of vibration exposure.

Annual sales data were obtained from all the companies' clinics: OHS 1 and 2 provided data from 2016 to 2022 and OHS 3 from 2020 to 2022. Given that MCVs should be offered at least every third year, data from 2020 to 2022 were aggregated for OHS 1 and 2, and supplemented with corresponding data from OHS 3, to capture all exposed workers who participated in an MCV.

To assess trends in completed MCVs, annual data from OHS 1 and OHS 2 for 2016–2022 were analyzed in terms of absolute numbers.

To estimate the total number of MCVs performed in Sweden, the combined figure from the three largest OHS providers was multiplied by two, as the collected data are estimated to cover half of all MCVs performed in Sweden.

To address potential limitations in the sales data, a sensitivity analysis was conducted using two datasets from OHS 2. To assess the number of unique individuals, as compared to sold services, we used a larger set (1 January 2016–6 March 2020) with pseudonymized data. To identify misregistered MCVs, we used a smaller subset (6 March 2019–6 March 2020), as this dataset included data on individuals where an MCV was registered that did not exist in the medical records, indicating that no MCV was performed.

#### Number of exposed workers

To estimate the number of workers eligible for MCVs, we used biannual official Swedish governmental statistics data (18), collected via interviews commissioned by the Swedish Work Environment Agency (SWEA, in Swedish Arbetsmiljöverket). The interview includes a six-level Likert scale measuring hand-arm vibration exposure, from “Almost all the time” to “Never” with intermediate options of $\frac{3}{4}$, $\frac{1}{2}$, $\frac{1}{4}$, and 1/10 of the time. Data are categorized by occupation according to the Swedish adaptation of the ISCO classification (19) and include the number of workers in each occupation.

Using the Statistics Sweden data, we constructed two exposure estimates. The *EU-OSHA estimate* included all workers exposed to hand-arm vibrations for at least 25% of their work time, as recommended by the EU-OSHA evaluation report (14). The *Quantified exposure estimate* was based on data from a 2016 meta-analysis (7), which reported the proportion of workers exposed to over 2.5 m/s^2^ A(8) by occupation. For occupations with multiple studies, we calculated a mean exposure rate from the meta-analysis data (provided by the authors) and applied it to the corresponding workforce size in Sweden (18).

#### Participation rate

We divided the sales data of performed MCVs for the period 2020–2022 by the EU-OSHA estimate and the Quantified exposure estimate to calculate the participation rates.

#### Statistics

We used STATA MP 17.0 (StataCorp, Texas, USA) for the statistical calculations describing the populations.

### Qualitative study

#### Preconceptions

CA, with a background in Occupational Medicine, had a preconception based on clinical work with vibration-exposed workers. CA also had experience from discussions about the avoidance of MCVs and rehabilitation with trade unions and larger companies from OHS. FT, as a specialist in Family Medicine without specific clinical experience in HAVS, brought a different perspective. CH brought yet another perspective as a non-physician, being an occupational therapist with extensive experience as a researcher at the Division of Occupational and Environmental Medicine, Lund University.

#### Informants

To capture diverse perspectives, we purposively selected first-order informants from OHS 2 medical records, including workers from vibration-exposed sectors with varied occupations, company sizes, geographic locations, age cohorts, and immigrant backgrounds. Medical records that provided insights into reasons for non-attendance at MCVs were also examined to guide strategic selection. We included two groups: a primary group of individuals who participated in an MCV, and a secondary group of individuals who declined participation despite reporting HAVS symptoms in a survey. This survey is occasionally used as a pre-selection tool for MCVs, although such use does not legally comply with the provision. We considered it important not to omit those individuals that do not get proper MCVs.

We contacted 25 MCV attendees whereof 18 participated (M1–M18, M for MCV), five did not respond, and two declined. Of the 14 additional workers who declined an MCV despite being symptomatic in a survey, eight participated (S1–S8, S for Survey), two declined, and four did not respond. Reasons for non-participation were not systematically sought, though some cited time constraints. One interview was incomplete due to connection issues and was analyzed as such.

The sample included 1 woman and 25 men, reflecting prior MCV participation rates of 6% women (20). The mean age was 49 (median 51.5, range 26–67). Among the informants were two union safety representatives who attended MCVs and one in the screened group. Descriptive statistics for occupation and birth year are presented in Table 1.

**Table 1:** Occupational and birth year statistics of the informants.

Trades	*n* Attended MCV	*n* Screened
Earthworks	2	3
Deconstruction/demolition	1	1
Carpenters	7	3
Fitters	4	0
Concrete workers	3	1
Park ranger	1	0
Birth year		
1957–1959	2	1
1960–1969	7	2
1970–1979	5	2
1980–1989	2	2
1990–1999	2	1
**Total**	**18**	**8**

#### Data collection

Data were collected via 26 semi-structured telephone interviews in Swedish. After obtaining verbal informed consent, interviews began with the question: “*Why do you think individuals avoid medical check-ups for vibrations?*” Then the interviewers used an optional guide, starting with company and coworker-related questions before exploring personal choices to facilitate discussion of sensitive topics. Questions included:

*Has your company been doing MCVs for a long time?*

*Do you know anyone who has avoided participating?*

*What did you do (concerning MCV)?*

*Have you avoided MCVs or rehabilitation discussions? Why?*

The interviewers' (CA and FT) knowledge on the subject was shaped by a yet unpublished study involving employers and unions (including first-order informants). This led them to explore factors affecting participation in MCVs, such as economic concerns, professional pride, lack of education, fear, time constraints, team loyalty, perceived unimportance, company considerations, lack of offers, bullying, and macho culture. These factors were included in the optional interview guide. Subsequent interviews were shaped by earlier ones, aligning with the hermeneutical process.

Both interviewers are experienced specialist physicians trained in history taking and managing sensitive topics. To avoid ethical issues and bias, they avoided interviewing known patients. Transcription was performed in verbatim, including notations for non-verbal communication, e.g. laughter, from the digitally recorded phone call by Spoken Ltd., Stockholm, a company specializing in academic research.

### Qualitative analysis

For the analysis, we used a qualitative method adapted for primary health care settings as described by Malterud (21, 22). The structure was based on a model described by Giorgi (23). The analysis was then performed using a hermeneutical method as described by Westlund (24, 25).

First, all interview transcripts were read globally to obtain the notion of the whole. Next, CA and FT systematically read through the transcripts to identify meaning units and extract supporting quotations to a single document.

The analysis of this single document was conducted in several steps. First, the six categories found in the global reading were verified. Second, the meaning units (sentences or paragraphs) were subdivided into subcategories after content and context and supported with quotations to preserve informant voices. Third, the meaning units were condensed into the adequate sub-category. This process yielded nine categories, each comprising one to three subcategories (a total of 14), as presented in Table 2.

**Table 2:** A schematic description of categories and subcategories.

Category	*Subcategory*
Economic fears	Fear of being laid off
	Fear of losing a good income
Fears of occupational changes	Fear of reassignment in the rehabilitation process
	Fear of losing the pride of being a true professional
Relational fears	Fear of being a burden
	Fear of being bullied or becoming an outcast
	Fear of not being believed of being injured
Fear of change	Fear of change
It's needless	HAVS is incurable anyway
	The doctor doesn't do anything anyway
Practical reasons	It's a hassle going to the check-ups
	Someone else should take the responsibility
Lack of knowledge	Unawareness of work environmental legislation
	I thought it needed to be vibrate more
The diagnosis was forgotten	The diagnosis was forgotten
The employer doesn't offer MCVs	The employer doesn't offer MCVs

In a fourth step, each transcript was reviewed in a recontextualisation process to assess that the subcategories and categories were distinct, both in terms of whether the subcategories had enough support from the transcripts, and to see that the transcripts did not yield any more meaning information. Finally, we assessed data saturation using both inductive thematic saturation (emergence of new codes or themes) and data saturation (repetition of existing data) models (26). Original recordings were consulted when transcripts were unclear or to evaluate tone and non-verbal cues.

All authors reviewed and assessed the analysis, comparing results with both condensed texts and original transcripts to enhance trustworthiness. CH conducted an independent analysis at this stage, contributing revisions that were discussed and integrated. Findings were also discussed with relevant stakeholders throughout the process.

All data analysis was conducted in Swedish to preserve meaning, acknowledging that translation involves interpretation (27). Quotations were subsequently translated into English, pseudonymized, and numbered to ensure clarity and anonymity. No software apart from Excel and Word was used in the qualitative analysis. In order to enhance credibility, transparency, clarity and scientific rigor, we followed the COREQ 32-item checklist for reporting qualitative research (28).

### Ethical considerations

All research followed the Declaration of Helsinki, with approval from the Swedish Ethical Review Authority (ref. no. 2021-00152). The approval for the qualitative part of the study granted permission to use recorded verbal consent for the research and use of quotations after individual answers were anonymised to the point of non-recognition in the final article. The informants first received written information about the study and their rights as described in the ethical approval. The verbal informed consent was recorded following an opportunity to ask further questions. In the quantitative study, sales data contained no personal identifiers, and use of medical record data was approved under existing ethical clearance, following an opt-out model recommended for registry studies (29).

## Results—Quantitative

### Number of performed MCVs

The number of performed MCVs increased considerably in OHS 1 and 2 from 2016 to 2022 (from 751 to 3,470; Table 3). During the period 2020 to 2022, 11,643 MCVs were performed in the OHS 1, 2 and 3. The three companies constitute one half of the Swedish branch. We doubled this figure and thus estimate that 23,286 MCVs were performed in Sweden during this period.

**Table 3:** Total number of sold MCVs at the three largest OHS in Sweden.

Year	OHS 1	OHS 2	OHS 3	OHS 1,2 & 3
2016	442	309	n/a	n/a
2017	524	319	n/a	n/a
2018	1,342	549	n/a	n/a
2019	1,546	748	n/a	n/a
2020	2,021	600	59	2,680
2021	2,998	555	441	3,994
2022	2,608	862	1,499	4,969
**Total sold**	**10,515**	**3,314**	**1,999**	**11,643**

To compensate for multiple appointments, we analyzed the number of unique individuals. Data from OHS 2's medical records consisted of 1,616 sold services, whereof 1,544 were unique individuals (mean age 45 years, range 19–70, 95% men, Table 4). Hence, 4% were MCVs that were repeated in the same individual over the three years that constitute the longest allowed time between two MCVs. Adjusting for these 4% multiple visits resulted in a corrected estimate of 22,355 individuals undergoing MCV triannually.

**Table 4:** Number of sold medical check-ups in worklife for vibration exposure at OHS2 over 3 years (1 January 2017 until 31 December 2019).

Year	Unique individuals, *n*	Sold MCVs, *n*	Male/Female, *n* (% male)	Age, mean (SD) range
2017	309	319	304/15 (95%)	45 (13) 20–66
2018	529	549	526/23 (96%)	44 (13) 19–66
2019	706	748	703/45 (94%)	45 (13) 20–70
Total	1,544	1,616	1,533/83 (95%)	45 (13) 19–70

To account for sold but unperformed MCVs, we reviewed OHS 2 medical records from 6 March 2019 to 6 March 2020. Of these, 19 cases lacked corresponding MCV documentation, indicating a 97% correct registration rate. Misclassified services included medical check-ups for other exposures (e.g., quartz dust, *n* = 5), other physician appointments (*n* = 2), and one health check. Five cases involved non-attendance, and six had no medical records. Further adjusting the estimate for unperformed MCVs resulted in 21,684 individuals undergoing MCVs triannually.

The formula used for calculating the estimated number of performed MCVs in Sweden:


$$\begin{array}{l} \frac{\begin{array}{c} Number\;of\;sold\;MCVs\;\left(11,643\right)\;x\;Unique\;indviduals\;\left(0.96\right)\\ x\;Correctly\;classified\;\left(0.97\right) \end{array}}{\begin{array}{c} Share\;of\;market\;\left(0.5\right)\;x\;Compensation\;for\;triannual\\ system\;\left(3\right) \end{array}} \end{array}$$

### Number of exposed workers

In total, 5,058,500 individuals were working in Sweden in 2021 (18). To calculate the EU-OSHA estimate, we used data from Statistics Sweden, which reports that the percentage of individuals between 16 and 64 who were exposed to vibrations for at least 25% of their workday remained steady at about 8% between 2017 and 2021 (18). The same figure has been reported since the late 1990s (30). Thus, approximately 400,000 individuals should be eligible for an MCV.

The *Quantitative exposure estimate* of eligible workers was based on a meta-analysis (7), which reported the proportion of workers in various occupations exposed to levels exceeding 2.5 m/s^2^ A(8). These percentages were applied to occupational workforce data in each trade (18), resulting in an estimated 217,658 workers eligible for MCV (Table 5).

**Table 5:** The number of workers in Sweden multiplied by the percentage of workers exposed above 2.5 m/s^2^ A(8) in their respective occupation according to data from Nilsson et al. (7).

Occupation	ISCO (19)	Swedish workforce, *n*	% Exposure above 2.5 m/s^2^ A(8)	Exposed Swedish workers, *n* (calculated)
Forestry workers	6210	15,550	64 %	9,952
Building and related trades workers	*71*	171,199	60 %	102,719
Sheet and structural metal workers, moulders and welders	*721*	25,042	100 %	25,042
Machinery mechanics and repairers	*723*	59,422	23 %	13,667
Plant and machine operators and assemblers	*834*	41,070	100 %	41,070
Labourers in mining, construction, manufacturing and transport	*93*	25,208	100 %	25,208
Total		314,953		217,658

### Participation rate

To calculate participation rates, the 21,684 MCV participants (2020–2022) were divided by the *EU-OSHA* and *quantitative exposure estimates* of exposed workers (Table 6), resulting in participation rates of 5% and 10%, respectively.

**Table 6:** Participation rate estimated by dividing 21,684 performed MCVs during a 3-year period with the estimates of exposed workers.

Estimate	Description	*n*	Participation rate	Source
EU-OSHA	Individuals exposed to vibrations 25% of worktime	400,000	5 %	(18)
Quantitative exposure	Workers exposed above 2,5 m/s^2^ A(8) multiplied by the percentage of workers in their respective occupation	217,658	10 %	(7, 18)

For calculating the participation rates we used the following formula:


$$\begin{array}{l} \frac{\begin{array}{c} Estimated\;number\;of\;performed\;MCVs\;in\\ Sweden\;(21,684)\;\left(see\;formula\;above\right) \end{array}}{\begin{array}{c} Estimate\;of\;exposed\;workers\;(EU-OSHA\;\left(400,000\right)\;or\\ Quantitative\;exposure\;\left(217,658\right)) \end{array}} \end{array}$$

## Results—Qualitative

### Economic fears

There were *Economic fears* as a common theme for the “Fear of losing a good income” and the “Fear of being laid off”. Both being fears where the informant perceived a risk of losing the possibility to support themselves economically.

#### Fear of losing a good income

Many informants perceived potential income reduction as a serious threat, noting limited alternative occupations with comparable salaries that do not require extended education. Several suggested the need for financial support during transitions, unaware that such aid exists via Swedish Social Insurance Agency annuities. Injured individuals were also frequently reported to face a non-voluntary early retirement.

“*You're sitting there, making 15–20,000* [SEK] *less a month. Maybe you have to switch jobs, things are looking pretty rough*.” M9 Carpenter, team leader

#### Fear of being laid off

Nearly all informants expressed concern about the socio-economic consequences of a HAVS diagnosis, rather than the functional impairments associated with vibration injuries. Fear of job loss was prominent, often grounded in accounts of colleagues who lost employment following a diagnosis. Over one-third explicitly feared termination, particularly due to concerns about diminished employability with age.

“*It could make their lives harder if it comes out that they have these injuries. It might even end up with them having to change jobs. I have a colleague who got vibration injuries and had to start operating machines instead to get away from all those vibrating machines, and I know he's had some trouble finding work after that. There just weren't any jobs for him*.” S2 Earthworks worker

### Fears of occupational changes

*Fear of occupational changes* includes the Subcategories “Fear of reassignment in the rehabilitation process” and “Fear of losing the pride of being a true professional”. This category comprises of fears of losing occupational status, interesting tasks and benefits, and personal identity. It is a category that primarily concerns fears directed toward the personal sphere and the concept of self.

#### Fear of reassignment in the rehabilitation process

Many informants expressed fear of job reassignment, using terms such as “*stopped*,” “*driven away*”, and “*condemned*”. Reassigned roles, particularly office work or tasks involving computers, were often viewed as monotonous, low-status, and undesirable. Some valued opportunities, such as travel-related positions, were lost following reassignment. Avoiding participation in vocational rehabilitation (VR) was seen as a strategy to prevent such outcomes.

Workers' loyalty to their teams was reported to mitigate fears of job reassignment and termination. One older worker, initially hesitant to disclose injuries, was motivated to inform management upon realizing it could benefit younger colleagues.

“*But it's a game of tactics. I mean, our case, the one that stood out, when I was in with the doctor talking about what we should do, he said: This shouldn't happen to the young guys, so I'll have to take this*.” M15 Fitter and union representative, relocated

Relocated informants emphasized that successful VR depends on effective collaboration between employers and unions, with trust in the employer being essential. Breakdowns in this process have led to negative outcomes, including fear and a culture of silence. Trust that the employer will offer support following a HAVS diagnosis increases the likelihood of employees attending medical assessments.

“*It wouldn't have had that* [positive] *outcome if they had laid off our index case. Then everybody would have shut everything inside themselves. Nobody would have said anything*.” M15 Fitter and union representative, relocated

#### Fear of losing the pride of being a true professional

Some informants expressed pride in their competence and professional identity, fearing its loss if relocated to unfamiliar trades or industries. They preferred career transitions within the same industry, a sentiment less common among industrial workers. Unlike the general fear of reassignment, this concern reflects a deeper personal identity loss.

“*What you want to retrain for, that's the hard part. If you‘re a carpenter, you're a carpenter, and that's what you live from. (…) I'm actually proud to be a carpenter. It's a joy*.” M4 Carpenter

#### Fear of being a burden

Many informants express a desire not to be a burden, primarily to their employer due to the fear of termination, but also to their colleagues within the work team. Several informants report that both workers and supervisors are aware of exposure levels far exceeding safety limits, yet reducing vibration exposure is very often deprioritised in favor of task completion. Harmful tools and methods persist, as safer alternatives are viewed as impractical and production cost increases are considered unacceptable.

“*Yeah, it's about building bridges, and for that, you need a certain type of concrete, so we have to use vibrators. You can't get around that*.” M10 Concrete worker

#### Fear of being bullied or becoming an outcast

A prominent factor identified is the ridicule, social exclusion, and bullying experienced by individuals showing signs of HAVS or expressing concerns about developing it. Reported across all company sizes and trades, about a quarter of informants reported personal experiences of such behavior, which discouraged participation in MCVs and VR. Both colleagues and supervisors engaged in bullying. Informants described supervisors mocking injured workers in front of colleagues, creating fear of similar treatment and suggesting this behavior is a strategy to pressure injured workers into resigning, avoiding employer responsibility for rehabilitation. Additionally, discussing HAVS rehabilitation is viewed as shameful.

“*You don't want to involve the company in it. Because in a way, it feels a bit shameful, kind of stigmatized. You don't want to go down that path*.” M12 Earthworks worker

Cautionary measures and workplace adaptations for HAVS are often dismissed as “*silly*”, with workers expected to endure and “*push through*”. Many informants exhibiting shame, stigma, and bullying also embody this macho attitude, especially toward women and younger workers. Over half described a culture where early medical care is taboo, with older workers delaying treatment until symptoms are severe. However, this mentality is gradually shifting, notably with increased female presence in the industry.

“*We don't go to the GP 3 days a week because we have a sore finger, we just hope it heals. It's probably pretty stupid, really, but it's a culture that's still around*.” S3 Demolition worker

#### Fear of not being believed to be injured

Several informants express doubt about the possibility of having vibration-related injuries recognized as occupational injuries, leading to a fear of not being believed. Some informants specifically emphasized the need for their injuries to be documented, as they believe the injury becomes credible only when formally recorded.

“*I'd really like to have it documented so I can refer to it if there are more injuries or if I can't work full time or something like that*.” M18 Carpenter

### Fear of change

The last category of fears includes only one subcategory “Fear of change” and was accordingly identically named.

### Fear of change

A few informants specifically highlighted a fear of any change whatsoever, rather than specific concerns. This fear of worsening circumstances seems to drive workers to continue despite potential injuries and future problems, reflecting a deep apprehension about an uncertain future.

“*I think a lot of it is fear. The fear of change that we have as humans. Change is always tough! I'm good as I am. Yeah, and you don't really know what you're going to get. What's on the other side of the fence?*” M2 Earthworks construction worker

### It's needless

#### HAVS is incurable anyway

Many individuals believe that MCVs are ineffective, as HAVS is incurable and manageable only through reduced or eliminated vibration exposure. Consequently, workers avoid MCVs, knowing that the doctor can only offer the solution they wish to avoid, adaptation or termination, seemingly overlooking that the development of HAVS can be influenced.

“*It can't be cured, so you probably just ignore dealing with it*.” S1 Earthworks worker

Conversely, some informants desire knowledge of their injury status and potential interventions to maintain control.

#### The doctor doesn't do anything anyway

Several informants reported inadequate examinations and insufficient support following a HAVS diagnosis, resulting in frustration and mistrust toward OHS, sometimes perceived as solely profit-driven. They emphasized the need for clear guidance from OHS on work requirements and rehabilitation options for workplace adaptations and relocation, something many did not receive. Some were never offered a vocational rehabilitation meeting with employers and physicians, and investigations were often stalled due to unclear follow-up responsibilities.

“*Many people like me at my company have brought this up in their meetings, saying they have white fingers or have trouble when it's cold outside, but then it gets written down and ends up in the doctor's report, and then nothing happens*.” S3 Demolition worker

The lack of follow-up and rehabilitation at university hospital Occupational and Environmental Medicine (OEM) clinics was reported as a significant issue. The informants stated that by not engaging in individual rehabilitation, OEM clinics fail to fulfill their expected role, leaving employees with only documentation for insurance claims and forcing them to manage rehabilitation independently—an approach viewed as highly inadequate.

“*I've been to work in environmental health [sic] in CITY. But then it's like, you don't get anywhere. You have the injury, but then nothing progresses, so it just fades away. You ask if there's any help available, and stuff like that. But nothing happens... It's like, yeah, it's not important enough, it's not enough*.” M9 Carpenter, team leader

### Avoidance due to practical reasons

#### It's a hassle going to the check-ups

Informants reported avoiding MCVs due to time away from work and discomfort in stepping aside from duties, creating a conflict between maintaining workflow and attending the exams, despite the latter being once every 3 years. Similarly, to minimize downtime, workers often exceed recommended exposure limits, sometimes without employer awareness. Difficulties in contacting OHS were also noted, and many believed that conducting MCVs onsite, rather than at clinics, would significantly improve participation.

“*I know many of my colleagues are very loyal to their job and find it difficult to leave work, to stop in the middle of the day and hand over responsibility to someone else. They want to keep track of their tasks and do them themselves*.” S2 Earthworks worker

#### Someone else should take the responsibility

Some informants argued that responsibility for managing the process should rest with the employer and OHS, rather than relying on individuals to report issues. Informants expressed a desire for supervisors to monitor exposure, despite practical challenges, whilst simultaneously valuing autonomy from managerial oversight. Informants said that diagnoses and recommended VR adaptations often were neglected, especially following changes in management or worksite — frequent in the construction industry.

“*So, at that place, I was doing all the drilling in the walls because there were thousands of holes, and they said, ‘You're not supposed to do that.' But then when I get to the next place, I'm still doing those tasks anyway because it hasn't reached the manager or whatever you want to call it. We're always moving around, so there's always new people*.” M8 Carpenter

### Lack of knowledge

#### Unawareness of work environmental legislation

Many workers lack awareness of their legal rights related to vibration exposure, MCVs, and rehabilitation, although some report adequate understanding among both workers and employers. Invoking these rights is often perceived as unwelcome by employers and may potentially lead to negative repercussions.

“*Yeah, but I pretty much know what you're entitled to and the company's responsibilities, I know that. But yeah, I still think I would've been replaced or looked at sideways, you know*.” M12 Earthworks worker

Several informants were unaware they were entitled to an MCV and therefore did not participate. Many confused mandatory MCVs with general health check-ups provided through industry-union agreements, even if they had received both. Seven out of eight informants in the screening group were unaware they had been offered an MCV by the OHS and did not participate, despite records indicating they had declined. While some HAVS cases are identified during general check-ups and referred for MCVs, many informants believed integrating MCVs into regular health assessments would be more effective.

“This thing with blood pressure and sh^*^t. Then you could've done that test at the same time.” M10 Concrete worker

Few informants were aware that professional confidentiality prevents physicians from informing employers about injured workers, likely contributing to fewer VRs. A mandatory aptitude report was suggested as a solution.

#### I thought it needed to be vibrating more

Several informants reported that both workers and supervisors often lack awareness of the harmful effects of tools, particularly impact tools, and may not recognize symptoms of vibration-related injuries. This lack of awareness leads to the perception that MCVs are unnecessary.

“*I thought it needed to be vibrating more than what we use. That you'd need something like jackhammers – not the simple machines we use. That's why I was surprised*.” M17 Fitter and union representative

Many informants admitted to disregarding others' injuries until personally affected, often expressing surprise at the relative absence of symptoms despite objective or semi-objective neurological findings. For several, symptoms of HAVS were not recognized until late in its progression, often only connected to HAVS when pointed out and confirmed during an MCV.

“*I didn't feel anything at that moment. I thought everything was fine, that I was good and my hands were okay, but it was more serious than I thought*.” M16 Fitter

The chronic nature of injuries and the permanence of nerve damage are often unrecognized, leading to negative surprise. However, when properly acknowledged, this chronicity can strongly motivate individuals to initiate a VR process.

“*And then you can almost see in people's eyes how they think about it a little extra. It's not like a cold that goes away if you stop, once you've got the injury, you've got it*.” M1 Carpenter

Improved understanding of HAVS progression is reported to enhance participation in MCVs. At least half of the informants noted growing awareness on worksites, leading to better adherence to exposure regulations and efforts to reduce vibration exposure. Many expressed a need for improved exposure monitoring, clearer guidance on limits, and greater knowledge of labor rights and relevant legislation. However, most workers receive HAVS and tool-related education only after diagnosis at an MCV. Mandatory education, as specified in the provision, aids early symptom recognition. A strict adherence to the triennial MCV interval prevents the injuries from becoming a shock.

“*I just think that if you do it more often, you wouldn't get that shock every time. Going from being completely healthy to having vibration injuries happens if you only go once... I mean, if it's just a tendency and you notice a small, small increase, then you need to do something, and you have to think about it. Instead of it being all or nothing, like it is if you only go once every 10 or 15 years or something. You go in healthy, and then suddenly, you're sick the next time!*” M2 Earthworks construction worker

Informants recommended initiating HAVS education in schools and reinforcing it throughout workers' careers, especially at employment onset. They emphasized the importance of managers, particularly those with blue-collar backgrounds, and experienced workers sharing personal experiences with vibration-related injuries to encourage MCV attendance among younger employees. This strategy was viewed as effective in emphasizing key safety regulations and countering macho cultural attitudes.

### The diagnosis was forgotten

Symptoms and even confirmed HAVS cases are sometimes overlooked and no VR is conducted. A notable example involved an informant diagnosed with severe HAVS a decade ago who later forgot the diagnosis. A subsequent check-up reconfirmed it, underscoring OEM clinics' failure to ensure rehabilitation.

“*Yeah, I had a check-up. When was this? Maybe three and a half, three years ago. But 10, 12 years ago, I had a check-up with someone at the hospital. And he told me, ‘You have to keep an eye on it yourself.' But... you forget about it*.” M14 Fitter, relocated

### The employer doesn't offer MCVs

In the group that attended an MCV, most informants noted that too few workers are offered medical check-ups, and even fewer are offered three-party meetings after findings or diagnosis of HAVS.

“*Yeah, I'd be really surprised by that. I've worked with this my whole life, but I've never been to a check-up just for vibration injuries. No, that's never happened*.” S4 Concrete worker

Several informants report having to request an MCV themselves, as it was not offered by the employer, despite legal requirements. Some struggled for years to gain access to one. The issue is not that workers avoid check-ups, but that they are rarely offered in the first place. Many informants noted that only certain employees, in particular older and those deemed more likely to be injured, are called for MCVs, despite the fact that all workers exposed to vibrations are eligible. Symptoms were also presented without subsequent suggestions for MCVs from either the employer or OHS. Several individuals with confirmed vibration-related injuries reported no rehabilitation measures or assistance in reporting the injury as work-related, which is illegal. Over half of the workers mentioned that MCVs are only offered after an injury occurs, requiring someone to be aware of their rights and brave enough to ask for an MCV. Many avoid doing so due to shame, bullying, macho culture, and fear of repercussions, leading to delayed care.

“I don't think many people take the initiative themselves. I don't think I would have done it either, not until it might've been too late.” M16 Fitter, relocated

## Discussion

### Qualitative methodological aspects

Ahmed (31) identifies four components for trustworthiness in qualitative research: *Credibility, Transferability, Dependability*, and *Confirmability*. Credibility was addressed through reflexive practices, where interviewers critically examined their preconceptions, and findings were reviewed with workers, union representatives, delegates, and employers (22). Transferability was supported by saturation analysis (26) and purposive sampling to maximize heterogeneity, complemented by thick descriptions and informant voices (32), ensuring transferability to Swedish, and potentially EU, contexts. Dependability was enhanced through transparent reporting of research procedures and reflection on methodological choices, including attention to negative or contradictory cases, such as informants expressing distrust in physicians. Confirmability was strengthened via discussions of findings with stakeholders and experts in OHS, OEM, and the Swedish Work Environment Agency, with substantial agreement across groups. The use of recorded oral consent after an opportunity to ask questions was reported back by one informant to be positive and important due to the occurrence of difficulties reading longer texts and dyslexia in the target population.

### Discussion of qualitative results

Our most important findings were that several different kinds of fear made injured workers abstain from MCVs, often in conjunction with a lack of knowledge about HAVS, vibration exposure, and legislation. Our results suggest that increased awareness leads to improvements in reducing workers' exposure levels, greater interest in MCVs, and enhanced willingness to adopt behavioral changes. The biggest reason for the low participation rate in the MCVs is, in all likelihood, that the employers far too seldom offer the mandatory MCVs to the exposed workers.

#### Avoidance due to fear

Fear was identified by all informants as a powerful driver of behavior. We found four different kinds of fear that in theory could be sorted under one super-category. Notably, fear of socio-economic consequences, such as job loss, reduced income, and fear of occupational changes such as loss of professional identity, outweighed concern about the illness itself. This contrasts with the substantial functional impact of HAVS, with two-thirds of patients reporting difficulty with basic tasks such as dressing (33), and quality of life (QoL) declining in correlation with HAVS severity (34). Additionally, the earlier onset of the sensorineural symptoms (4) has a more substantial impact on QoL than other HAVS symptoms (35).

The fear of reassignment, particularly away from manual labor to office work, might reflect both a macho culture among blue-collar workers and a perceived lack of aptitude in office tasks due to prior negative school experiences. Additionally, severe HAVS impairs fine motor skills (36), affecting tasks like typing, writing, and prolonged sitting, which complicates retraining, as previously noted in a Swedish study on women (37), underscoring the need for early intervention. This study also highlights that HAVS leads to a loss of professional pride and identity, contributing to perceived lower status. This sentiment, shared by male construction workers in the present study, is presumably far more generalized and is likely to be a key factor behind non-participation in MCVs.

Bullying of individuals with HAVS or concerns about developing the condition was reported across all company sizes, involving both colleagues and managers. In feedback on the results, the construction workers' union identified this as a prevalent issue, potentially contributing to the previously reported higher suicide risk among male construction workers, linked to stigma around mental health, physical illness, bullying, and macho culture (38). A Swedish study highlighted the role of management in creating a supportive environment, where employees can discuss concerns and negative behaviors are not tolerated, with OHS support being crucial (39).

The social environment of our informants was largely shaped by a declining macho culture, with some individuals acknowledging its negative impact. Machismo and male identity have been found to be key factors in the challenges faced by individuals with HAVS, including fears of unemployment, financial strain, and loss of male identity (16). Similarly, male HAVS patients in northern England reported frustration, isolation, and social withdrawal due to embarrassment, suggesting the transferability of these experiences to, at least, other European contexts.

An interesting finding was how work was generally prioritized over health, with significant anxiety about being a burden to colleagues and employers. This, in combination with the culture of silence around HAVS and perceived economic priorities outweighing safety, is something to be remedied, as this obstructs most systematic work with the work environment. This should be a prioritized area for unions and managers by clearly stating that safety is imperative and should cost money, thus fostering a culture of safety in the workplace. Further, the cultural norm against seeking medical care before a condition is confirmed is another finding that should be mitigated by collaboration between unions and employers with the aid of OHS. Such collaboration, in combination with education, is also essential for mitigating fear.

#### Needlessness and practical reasons

The category of needlessness had a fatalistic tone, with informants believing that little could improve, bordering on the fear of change. In some cases, this view overlapped with the sub-category of lack of knowledge, as some misconceptions stemmed from ignorance of facts.

Some informants reported limited access to OHS, with visits often perceived as unnecessary due to subsequent referrals to general practitioners. This confusion may stem from a lack of clarity regarding the division of responsibilities between public health services and employers contracting for OHS. Clarifying the role and responsibilities of OHS could help address this issue. The situation is further complicated by misalignment between employers and OHS on the scope of the latter's mandate, intensifying workers' feelings of inadequate support.

General Practitioners (GP) lack expertise in HAVS and often miss the workplace etiology, leading to doctors' delays in further referral with aggravated injuries (17), calling for increased education for GPs. For employees at companies without an OHS contract—approximately 40% of workers in Sweden (40)—the OEM clinics are often the only option for diagnosis and competent VR support. Thus, no VR takes place when the OEM clinics fail to complete their presumed role by not participating in VR, highlighting the need for continued support from either OEM clinics or a continuous process where another stakeholder is made sure to continue the VR-process.

Many workers offered an MCV express skepticism about its necessity, largely due to the gradual, mild symptoms of Hand-Arm Vibration Syndrome (HAVS), which are initially not perceived as severe or dangerous. This aligns with findings from Canada, where 70% of workers delayed seeking medical aid, believing the symptoms had a natural cause, and 57% did not consider them serious enough (17).

Increasing participation in medical examinations could be achieved by facilitating on-site screenings or offering scheduled appointments by letter. It was noted that information on diagnoses and adjustments often disappeared when construction workers were transferred to different sites or employers. In such cases, it was left to the individual to communicate the details. This highlights the need for a system to monitor vibration-related injuries and ensure necessary VR adaptations are consistently applied.

#### Lack of knowledge

Informants‘ underestimation of exposure and symptoms, as well as forgetting about injuries, may stem from a lack of knowledge about HAVS, regulations, and misinterpretations of symptoms and physicians' advice. Bodley reported that workers minimized their symptoms progressing until disability, possibly due to a lack of understanding of the progressive and irreversible nature of HAVS (17). Ayers and Foreshaw suggest that the underestimation of physical symptoms, combined with fears of unemployment and insufficient support, reduces psychological well-being and increases stress for HAVS patients and their families. This may result in patients minimizing the physical effects of HAVS to avoid losing their jobs (16). Our results suggest that multiple appointments to the OHS that monitor injury progression and provide information are reported to make patients link their symptoms to HAVS and induce willingness to participate in VR.

In addition, information on regulations, exposure risks, vibration-related injuries, and available preventive and support measures must be communicated more effectively. Such communication should address workers' fears and reduce uncertainty, which contributes to anxiety. Additionally, alignment between employers and unions in delivering a consistent message significantly enhances participation. Lack of awareness of physicians' professional secrecy and alternative pathways within the VR process often results in inaction. Therefore, the mandatory education on these topics should begin in schools and continue throughout workers' careers.

#### No one has offered me an MCV

Limited access to MCVs, typically restricted to high-risk individuals, was identified as the primary reason for low worker participation. This reflects a failure to comply with regulatory provisions. A significant lack of knowledge about vibration-related injuries and legal requirements among employers contributes to this issue. In this context, educational efforts should target employers to increase the offering of MCVs.

Legally, there is strong support for mandatory MCVs by both employers and employees. An introduction of an aptitude report is expected to improve participation rates but reduce autonomy in the decision. Given the discrepancy between self-reported symptoms and objective functional impairment (41, 42), reliance on questionnaires alone is likely to fail to detect early-stage HAVS. Thus, only physician-led examinations should remain to ensure credibility. Union representatives further emphasize the need for more frequent and rigorous inspections by the Swedish Work Environment Authority (SWEA) to strengthen workplace health and safety and to oversee the employers' adherence to all mandatory regulations.

Listed in Table 7 is a short recollection of some measures with the implications to improve the likelihood that the employer offers an MCV and that the employee will attend the MCV and a possible subsequent VR. These measures derive both from informant's suggestions in the results section as well as conclusions drawn by the authors based on these results.

**Table 7:** Suggestions to mitigate low participation with their proposed mechanisms and implications.

Counter measure	By	Mechanisms and implications
Knowledge on surveillance program, VR-processes, societal safety nets, and HAVS	Employee	Reduction of fears, alternative VRs proposed. Reduces confusion with other check-ups. Removes logic fallacy that HAVS is incurable so nothing could be done.
Knowledge about exposure	All	Reduction of underestimation of getting injured. Knowledge of action limit as inclusion for MCV.
Knowledge on professional secrecy and roles of OHS	All	Reduction of ignorance due to data not being transferred. Adequate expectations on the OHS reducing fears. Improving VRs.
Union and employer sharing a common knowledge on HAVS and the parts' responsibilities	Union and employer	Increased collaboration and less distrust reduce fears. Shared view on risks and possibilities. MCVs are offered to all eligible employees. Less misinterpretations of provisions. VRs with more possibilities.
Information by trustworthy blue-collar workers	Employer	Reduction of fears and distrust. Countering toxic culture and ignorance.
Reassignment in the same branch	Union and employer	Reducing fears, primarily of losing professional pride and fear of reassignment.
Fostering a culture of safety	Union an Employer	People can discuss their concerns which reduces fear and cultures of toxicity and silence. Information that costs for safety measures are acceptable. Reduction of fear of being a burden
Increase of female workers	Employer	Reduces fear of bullying, machismo, and a toxic culture.
Documents on findings of HAVS and VR-adaptations kept by employer	Employer and OHS	Facilitates monitoring of exposure and information to individuals at risk. Diagnoses are not forgotten Reduces risk of adaptations not being passed on to next worksite. Reminds employer to book MCVs.
OEM process to transfer HAVS cases for VR	OEM	VR takes place and diagnoses are not forgotten. Reduction of fears and mistrust.
Physician's medical examination every MCV	Employer and OHS	Reduction in risk of missing early signs due to ignorance reducing underestimation of symptoms.
MCV performed at work site with proper written invitation	Employer and OHS	Reduces the hassle of booking. Stresses the importance of the MCVs. No action needed by worker.
Multiple appointments with short intervals after findings of HAVS	OHS	Reduces risk of underestimation, less misinterpretation of physician's recommendations. Monitoring of injury progress.
Mandatory aptitude reports	SWEA	No misinterpretations of the provisions. Increased importance. No one can work without MCV.
More inspections by SWEA	SWEA	Increased importance. A wish to do the right thing. Increased knowledge. Economic incentive.
Comprehensive process by OHS	OHS	All of the above.

### Discussion—Quantitative part

Our most important findings were that only a minority (5%−10 %) of the eligible workers exposed to vibrations participated in an MCV. Approximately 7,000 MCVs are estimated to be performed annually, a number that needs to increase dramatically to prevent workers from developing HAVS. Positively, the number of MCVs has increased almost fivefold between 2016 and 2022.

These findings should be relevant for OHS personnel, policymakers, and labor market stakeholders in affected sectors, as they highlight the insufficient implementation of MCVs. They are also of interest across EU member states, given that the medical surveillance program is mandated by an EU directive that also included the UK.

Only one prior study on vibration-related medical surveillance was identified. In this UK study, just 18% of workers in high-risk industries had undergone HAVS examinations, with smaller companies being least likely to provide health surveillance (43), a finding echoed by our study. This disparity was attributed to larger firms having dedicated personnel (e.g., HSAs, occupational health engineers, HR) responsible for such matters.

From a methodological perspective, a clear limitation is the exclusion of some Swedish OHS providers. However, the three included providers represent a substantial share of the market and serve industries with high vibration exposure, covering all occupations listed in Table 5. They offer the full range of services expected of OHS providers, including engineering expertise for measuring hand–arm vibration exposure. In contrast, some smaller OHS providers report no vibration-exposed workers among their clients, either due to the nature of the industries served or because employers do not offer MCVs. This could result in low physician competence in smaller providers as they might completely lack previous experience of handling MCVs. However, the problem that OHS physicians lack knowledge on HAVS has previously been shown to be the case several for several OHS-companies, including but not limited to the large OHS providing data in this study (20). Since we haven't been able to obtain data from other providers, this real-world data is the best available. With respect to this we consider the included OHS providers to be sufficiently representative to support estimates generalisable to Sweden. Given that the three included OHS account for approximately 50% of the market and predominantly serve vibration-exposed sectors, the assumption that they conduct around half of all MCVs is, if anything, conservative, in which case even fewer MCVs are performed.

We chose to use official Swedish governmental statistics, based on self-reported data, to reach the total number of exposed workers. A Swedish report indicates that self-reported estimates tend to overstate actual trigger times (44). However, the same report concluded that when evaluating risk for injuries, the exposure to vibrations should be based on trigger times estimated by the workers. They derived this from the fact that the action and limit values themselves are based on self-estimates and using more correctly measured trigger times would thus underestimate the risk of injuries, undermining the purpose of the MCV.

Analysis of sales data from OHS 2 suggests that approximately 3% of entries were misclassified as MCVs—a minor deviation that supports the validity of using sales data in this study, and that we corrected. We compensated for both these misclassifications as well as for the number of multiple visits, which we deem gives a more valid result. However, some MCVs may have been recorded under other service categories, which could not be assessed in the material. Overall, we find that the clinical context lends the data high ecological validity. Additionally, the dataset from Statistics Sweden is ISO-certified (20252:2012) and considered high quality.

## Conclusions

Participation in the Swedish medical surveillance system for vibration exposure remains low, with only around 5%−10% of vibration-exposed workers attending between 2020 and 2022. Although rates are gradually increasing, significantly higher participation is needed to prevent HAVS.

As the main issue is employers' failure to offer MCVs, ensuring universal access is critical. Additional factors include fear, limited knowledge, and workplace culture, which create short-term incentives to avoid both MCVs and vocational rehabilitation, which should be supported by targeted information. Our findings indicate that increased awareness by all stakeholders can reduce exposure, boost interest in MCVs, address workplace culture and fear, and promote behavioral change. To improve participation and reduce HAVS-related disability, these efforts must be part of a coordinated process with clearly defined responsibilities for all stakeholders.

## Author's note

Carl Antonson is a Swedish male, MD and PhD, and a certified CBT-therapist. He works as a GP and occupational physician and specialises in both family medicine and occupational medicine. In his work as an occupational physician, he is responsible for medical check-ups as well as vocational rehabilitation for patients with HAVS. He has previously done research on stress and HAVS.

Carita Håkansson is a Swedish female, OT and PhD. She works as a researcher at the Division of Occupational and Environmental Medicine, Lund University and is an experienced researcher both in qualitative and quantitative research. She has mainly done research on stress, work-life balance, and psychosocial work environment, but has no experience with HAVS.

Frida Thorsén is a Swedish female, MD and PhD. She works as a GP and specialises in Family medicine, where HAVS may occur sporadically. She is doing research on stress and HAVS. at the Center for Primary Health Care Research at Lund University.

## Data Availability

The datasets presented in this article are not readily available because the complete and detailed individual data of all subjects cannot be publicly available for ethical and/or legal reasons due to compromising patient privacy, based on Swedish law. The National Ethical Committee (https://etikprovningsmyndigheten.se/en/) has imposed these restrictions. Data can be obtained after application and approval of the research project by the National Ethical Committee and by the data safety committee of the university. Requests to access the datasets should be directed to carl.antonson@med.lu.se.
